# *“The wondrous eyes of a new technology”*—a history of the early electroencephalography (EEG) of psychopathy, delinquency, and immorality

**DOI:** 10.3389/fnhum.2014.00232

**Published:** 2014-04-17

**Authors:** Felix Schirmann

**Affiliations:** Behavioral and Social Sciences, Theory and History of Psychology, University of GroningenGroningen, Netherlands

**Keywords:** electroencephalography, EEG, history, psychopathy, brain, neuroscience, morality

## Abstract

This article presents a history of the early electroencephalography (EEG) of psychopathy, delinquency, and immorality in Great Britain and the United States in the 1940s and 1950s. Then, EEG was a novel research tool that promised ground-breaking insights in psychiatry and criminology. Experts explored its potential regarding the diagnosis, classification, etiology, and treatment of unethical and unlawful persons. This line of research yielded tentative and inconsistent findings, which the experts attributed to methodological and theoretical shortcomings. Accordingly, the scientific community discussed the reliability, validity, and utility of EEG, and launched initiatives to calibrate and standardize the novel tool. The analysis shows that knowledge production, gauging of the research tool, and attempts to establish credibility for EEG in the study of immoral persons occurred simultaneously. The paper concludes with a reflection on the similarities between EEG and neuroimaging—the prime research tool in the current neuroscience of morality—and calls for a critical assessment of their potentials and limitations in the study of immorality and crime.

## Introduction

“Presumably prisons harbor many persons afflicted not with moral turpitude but with *disordered brain waves* which require chemical therapy, or, for the protection of society, eugenic prophylaxis.” (Lennox, 1942, p. 594, emphasis added)

In 1942, William Gordon Lennox, a pioneer of epilepsy research at Harvard Medical School, surmized that researching *disordered brain waves* could revolutionize the understanding of immorality. Lennox' anticipation was incited by a recently invented technology that visualized brain activity: electroencephalography (EEG). Approximately 70 years later, modern neuroscience has appropriated immorality and delinquency as viable objects of research. Especially the emergence of neuroimaging technology in the last two decades facilitated research on the good, the bad, and the brain. According to some, a revolution in understanding morality is on its way (Sinnott-Armstrong, 2008; Fumagalli and Priori, 2012). Both technologies—EEG and neuroimaging—have opened up new epistemic spaces, unlocked new phenomena, spurred research, generated promises of scientific progress, encountered methodological problems, and attracted diverse criticism (Borck, 2005a; Schleim and Roiser, 2009; Choudhury and Slaby, 2012; Rose and Abi-Rached, 2013). Both technologies afforded unprecedented empirical access to the living brains of wrongdoers and generated hopes for imminent solutions for the intractable problem of immorality. This resemblance is worth exploring.

The hype surrounding both technologies has to be understood in historical, social, political context for it influenced their potential for deployment as well as their capability to provide solutions for social problems (Hayward, 2001; Borck, 2005a; Alder, 2007; Ortega and Vidal, 2011; Bunn, 2012). While neuroimaging research has received copious scholarly attention (Dumit, 2004; Littlefield, 2009; Choudhury and Slaby, 2012), the history of EEG of psychopathy, delinquency, and immorality has not been addressed with sufficient detail yet. Historical work in the area only touches upon the issue (Borck, 2005a; Rafter, 2008; Verplaetse, 2009; Rose and Abi-Rached, 2013; Pickersgill, 2014). How were “the wondrous eyes of a new technology” (Syndulko, 1978, p. 145) constructed, used, and appraised? Answering this question provides valuable insights into the potentials and limitations of brain science—then and now.

In this paper, I present a history of early EEG research on psychopathy, delinquency, and immorality in Great Britain and the United States in the 1940s and 1950s. Though different histories could be written, my focus is on the experts' appraisal of the new research tool, the associated problems and proposed solutions. The paper commences with a sketch of the complex history of somatic immorality and the state of Anglo Saxon psychiatry in the 1940s. Subsequently, the heterogeneous concept of psychopathy along with its moral connotation and the emergence of EEG as a tool for psychiatric research are described. The body of the paper specifies the role of EEG in the diagnosis, classification, etiology, and treatment of unethical and unlawful persons. Though initially experts had high hopes for the new technology in this field, they soon realized that its use involved methodological problems. EEG, then, was a novel, unstandardized research tool still under development. The experts were uncertain and at odds regarding its proper application and the interpretation and utilization of the generated data. Uncertainty and dissension pervaded EEG research on misdemeanants. The research community tried to manage this uncertainty through developing research standards, devising criteria for the interpretation of data, refining the technology, discussing the validity of EEG, and acknowledging the tentativeness of their findings. The analysis shows that knowledge production, calibration of the research tool, and the establishment of credibility of the new technology in the study of immoral persons occurred simultaneously. The paper concludes with a reflection on the past and present of the brain science of morality, arguing that persistent methodological and theoretical problems already inherent in early EEG research call the merit of modern neuroimaging technology into question.

## Historical context

### Somatic immorality—a long history

Immorality has to be understood in context—as well as corresponding mental disorder concepts and their putative biological manifestations. Ideas on a hereditary, physiological, or neurological basis for anti-sociality, violence, and crime have a long and multifarious history (Rafter, 2008). In the course of the 19th century, bio-medical theories on the genesis and proliferation of immorality emerged from early psychiatry, criminal anthropology, and Social Darwinism. Diverse moral transgressions (ranging from promiscuity over alcoholism to murder) were re-conceptualized and treated as biological disease. The focus on criminals' bodies and brains gave rise to a new class of medical experts, new discourses on guilt, and new options for policing and controlling badness. Against this backdrop, bio-medicine offered solutions for the management of society, e.g., via eugenics (Smith, 1981; Richards, 1987; Wiener, 1990; Becker and Wetzell, 2006; Schirmann, 2013a). Although bio-medical views on immorality and crime had been continuously criticized on scientific, social, and political grounds, they still constituted an explanatory resource within British and American psychiatry in the 1940s and 1950s. At that time, the field was dominated by biological and psychodynamic views, which often mixed in the description of anti-sociality (Shorter, 1997; Wallace and Gach, 2008; Pickersgill, 2010). Various classification systems for mental disorder co-existed. Experts advocated different nosologies and etiologies, reflecting their training and theoretical commitments. The publication of the first *Diagnostic and Statistical Manual of the American Psychiatric Association* (DSM) in 1952 was an attempt to homogenize classification. Among others, the definition of one particular mental disorder was arduous: psychopathy.

### The concept of psychopathy—a wastebasket with moral overtones

“It is a confusing term; it is misleading, and it means nothing.” [Dr. James J. Ryan at a meeting of the Philadelphia Psychiatric Society (see Matthews, 1949, p. 872)]

Who is a psychopath? What is psychopathy? These questions were indelibly intertwined. Identifying psychopaths required a definition of the condition. In the 1940s, definitions were plentiful—and contradictory. Since its origination in nineteenth century Germany, the term *psychopathic* had acquired multiple meanings, designating a host of different people and mental conditions (Werlinder, 1978; Sass and Herpertz, 1995; Wetzell, 2000). The notorious ambiguity of the concept expressed itself in multiple manifestations (e.g., psychopathic inferiority, psychopathic personality, constitutional psychopathic state). In 1923, at a symposium initiated by Benjamin Karpman, who would later become one of Americas leading experts on psychopathy, the concept was called “a wastebasket into which all sorts of things have been thrown” (cited in Werlinder, 1978, p. 147). In the following decades, the psychiatric community on both sides of the Atlantic almost unanimously criticized the equivocality of the concept (Partridge, 1930; Curran and Mallinson, 1944; Preu, 1944; Gurvitz, 1951). Some psychiatrists even called for its abolishment: in 1948, Karpman stated that psychopathy has become so diluted that “what remains is a myth and is a non-existent entity” (Karpman, 1948, p. 527).

The vacuity of the concept in the 1930s, 1940s, and 1950s called for clarification. Several definitions, classifications, and typologies emerged (for bibliographies, see Maughs, 1941, 1955; Hare and Hare, 1967). For example in Great Britain, Henderson (1939) proposed three types of psychopaths: a predominantly aggressive, a predominantly passive or inadequate, and a predominantly creative type. In the United States, Partridge's (1930) sociopath (codified in the first DSM in 1952) as well as Cleckley's (1955/1941) and Karpman's (e.g., 1947, 1948) work on the concept were seminal (for overviews, see Sass and Herpertz, 1995; Hervé, 2007). These attempted conceptual refinements occurred simultaneously with an increment in research: whereas 28 articles in English appeared on psychopathy from 1930 to 1940, 171 articles were published from 1940 to 1955 (see also Cason, 1948; see McCord and McCord, 1956, p. 9). Apparently the concept attracted researchers' attention; but the question remained: what characterized the psychopath?

Despite ambiguity, many definitions converged on the presence of persistent anti-social, immoral, and illegal conduct (Curran and Mallinson, 1944; Preu, 1944; Darling, 1945; East, 1945). Several classifications of psychopathic personalities contained a distinct anti-social or amoral type, such as East's (1945) *ethical aberrant personalities* or Karpman's (1948) *anethopaths*. These persons' callous, egotistic, aggressive, indecent, and unprincipled behavior appeared unalterable. Karpman (1947) called their unreceptiveness to ethical training, treatment, and punishment *moral agenesis*. “In general,” Kennard (1953, p. 101) wrote, “the psychopathic personality is said to be the individual who has little or no moral sense or social responsibility and who, in consequence, performs acts which are amoral or antisocial without sufficient sense of guilt to restrain future performance.” These character traits often manifested in crime. Experts noted the overlap between psychopathy and delinquency. Some even stated that delinquency was a criterion for the diagnosis (Preu, 1944; McCord and McCord, 1956; Craft, 1966). Hence, differentiating between the mentally disordered psychopath and the mere criminal was difficult; a momentous difference with regard to legal responsibility (Gibbens, 1951; Conrad, 1959; Ward, 2010). Contributing to these difficulties was the obscurity of the causes of psychopathy, delinquency, and immorality. An editorial to a special issue on the psychopathic offender in the *British Journal of Delinquency* in 1951 stated that “our understanding of psychopathy is still rudimentary and our researches wretchedly inadequate” (Anonymous, 1951, p. 77). Neurological evidence was particularly scarce: the functioning of the living brain of psychopaths was inscrutable. This situation changed dramatically when a new research tool became available in the 1930s.

### The emergence of electroencephalography (EEG)

Electroencephalography—the recording of the brain's electrical activity—had a slow start. Certain electrical properties of the nervous system had been known since the late eighteenth century, yet only in the 1920s the German neurologist Hans Berger constructed a device that registered an electrical rhythm on the human scalp. In the 1930s, Edgar Adrian replicated and popularized Berger's findings, stimulating the use of EEG as research tool in neurology and psychiatry (Brazier, 1961; Borck, 2005a). The new technology spread from Germany, to Great Britain, and the United States along different trajectories in the respective national contexts. The initial slow start was followed by a massive growth “[a]s the field of EEG expanded after World War II with the speed and vigor of a prairie fire” (Adrian, 1963/1950; Knott, 2009, p. 155). In the 1940s, various EEG laboratories emerged, congresses were held, an EEG journal was launched, and professional societies originated (e.g., the American Electroencephalographic Society was founded in 1946) (Niedermeyer and Lopes da Silva, 1987; Stone and Hughes, 2013). Experts valued the EEG signal as reliable indicator of brain function. The new technology soon proved great utility in the study of epilepsy and the localization of tumors. What other riddles might EEG help to solve? What hidden meaning was encoded in the changing amplitude and frequency of the brain waves? Researchers began to explore the potential of the new technology, applying it to all sorts of neurological, psychiatric, and psychological phenomena. In his marvelous book on the cultural history of electroencephalography, Borck (2005a, p. 232) described the situation as follows: “with the scientific recognition of the EEG as valid parameter for brain function the entirety of human life from procreation to death could count as object for electroencephalographic investigation, and indeed almost no human activity was left without a representation in form of an EEG-curve.” Starting in the late 1930s, EEG studies on intelligence, personality, psychosis, homosexuality, and peptic ulcers began appearing (Davis and Davis, 1939; Lindsley, 1944; Ellingson, 1954). Around the same time, “the wondrous eyes” of EEG caught sight of psychopathy, delinquency, and immorality.

## The EEG of psychopathy, delinquency, and immorality in the 1940s and 1950s

### The inception: early EEG traces of unwanted behavior and a sprouting hope

In 1938, a study with the title “Electroencephalographic analyses of behavior problem children” appeared in the American Journal of Psychiatry. The authors claimed that the EEG records of so-called behavior problem children deviated from those of normal children (Jasper et al., 1938). This was one of the first studies that identified traces of unwanted behavior in the electrical activity of the nervous system. What is more, the logic of the conducted comparison exemplified a seminal assumption of the study: behavioral aberration correspondents to abnormal brain waves; deviant people have deviant brain functioning. This momentous speculation guided research in the years to come. Soon more experts conducted EEG research on behavior problem children and delinquent boys whose abnormal brain waves made it seem likely that “[t]hese children will furnish some of the […] psychopaths of their generation” (Lindsley and Cutts, 1940; Brown and Solomon, 1942, p. 264; Gallagher et al., 1942; Gottlieb et al., 1945; Michaels, 1945). Accordingly, psychopaths and criminals attracted experts' attention in the 1940s. East and west of the Atlantic, researchers attached electrodes to the scalps of inmates of specialized asylums and prisons. In Great Britain, Denis Hill, Desmond Pond, David Stafford-Clarke, Trevor Charles Noel Gibbens—all with ties to the Maudsley Hospital in London—formed the forefront, with William Grey Walter and others contributing and commenting. In the United States, initially Frederic and Erna Gibbs at Harvard (Cambridge, Massachusetts), John Knott and colleagues at the Iowa Psychopathic Hospital (Iowa City, Iowa), and Daniel Silverman at the Medical Center for Federal Prisoners (Springfield, Missouri) studied misdemeanants' brain waves, inspiring others to follow them.

Researchers had high hopes for the new, promising tool. The enthusiasm reflected in Lennox' remark about “moral turpitude” not being sin, but disease reflected by “disordered brain waves” (see above) reverberated through several early EEG publications. The hopes clustered around four themes: diagnosis, classification, etiology, and treatment of the unethical and unlawful. Improving diagnosis could be attained by EEG's capacity to provide “objective data” (Hill and Sargant, 1943, p. 527) which might purge subjectivity from diagnosis (Gibbens et al., 1955). Furthermore, EEG could aid to elucidate biological nuances in psychopathic personalities that evaded the eye of the psychiatrist (Diethelm and Simons, 1946). With regard to etiology, EEG could rule out tumors or epilepsy as causes for immoral behavior and could illuminate an organic origin of psychopathy and delinquency (Silverman, 1947). Also, EEG established “new vistas for therapy” (Silverman, 1943, p. 30) which promised improved therapeutic success (Knott and Gottlieb, 1943). A few researchers even speculated on EEG's power in determining criminal responsibility (Stafford-Clark and Taylor, 1949; Conrad, 1959). Yet, others accentuated the limited value of EEG in psychiatry (Lindsley, 1944; Walter, 1944). But even amongst the critics, there were hopes that “when the new techniques are perfected” (Walter, 1944, p. 73), psychiatry and criminology might profit (see also Levy and Kennard, 1953). In general, hopes were fraught with uncertainty: in the 1940s, nobody knew whether EEG could deliver on its promise to define, classify, explain, and treat psychopathy, delinquency, and immorality.

### Defining the “elephant”: EEG in diagnosis and classification

“I can't define an elephant; but I know one when I see one.” (Curran and Mallinson, 1944, p. 266)

When the first brain waves of immoral people unfurled, informed judgments on their composition, their supposed oddity, and their meaning were needed. The criterion of choice was comparative: experts gauged the deviation between EEG curves of abnormal and normal people. The deviation was then interpreted as neurological trace of unethical behavior. Such traces were abundant: Hill and Watterson (1942) reported EEG abnormalities in aggressive psychopaths. Knott and Gottlieb (1943, 1944) stated that about half of the psychopathic personalities in their sample had abnormal EEG. Silverman (1943) found approximately the same abnormality ratio in psychopathic criminals. Something seemed out of tune in the brain rhythms of psychopaths. Moreover, there appeared to be distinct degrees of abnormality for different psychopathic sub-groups. For example, Silverman (1943) reported diverging percentages of abnormality for hostile, hedonistic, inadequate, and homosexual psychopaths. However, EEG did not detect deviant rhythms in mere criminals (Hill and Watterson, 1942; Silverman, 1944a). Robbery, larceny, murder, sex offences, assault and battery were untraceable in the brain waves—as a whole and as individual categories (Gibbs et al., 1945). Nevertheless, dividing psychiatric or legal categories into sub-classes in order to search for distinct EEG abnormalities was common. Stafford-Clark and Taylor (1949) split the offences of murderers into incidental, clearly motivated, apparently motiveless, sexually motivated, and driven by insanity. Their results were somewhat puzzling: motiveless murder was clearly associated with an abnormal EEG whereas the abnormality was negligible for other types of murder. Seemingly, the EEG could be of use in discerning different types of psychopaths and criminals; but could the new technology aid in delineating psychopaths from criminals? Starting in 1948, a major British study investigated the psychopath in prison with the aim of deciding this question and improving the diagnostic criteria for psychopathy (Stafford-Clark et al., 1951; Hill and Pond, 1952; Gibbens et al., 1955, 1959). Gibbens and colleagues collected psychological and electrophysiological evidence on a fairly constant sample of incarcerated psychopaths for several years, finding—among other things—that EEG abnormality was “four times as frequently in the psychopaths as in the controls” (Gibbens et al., 1955, p. 131). In a similar vein, an American study suggested different degrees of EEG abnormality to correlate with type and severity of crime (Levy and Kennard, 1953).

However, abnormality was a coarse criterion. It was crucially dependent on what was deemed normal (see Analysis below). Some researchers responded to this caveat by tightening the criteria for abnormality on the behavioral and the electrophysiological level, resulting in negligible differences between psychopaths and controls (Simon et al., 1946). Generally, more refined criteria for the classification of brain waves could aid to improve accuracy and consistency in the nascent discipline. What exactly did abnormality consist in? And, were there characteristic aspects of the EEG signal that signified badness? In general, brain waves could be too fast or too slow (Hill and Watterson, 1942). The early studies had indicated slowness—that is, low frequency—as conspicuous aspect in EEG recordings of behavior problem children. In this context, particularly the theta rhythm (4–7 cycles per second) was a promising candidate. Several studies reported slow activity in general or theta activity in particular in the EEG of psychopaths and criminals. For example, Ostow and Ostow (1946) noted the strong correlation between specific slow activity and antisocial behavior in a diverse group of inmates (Knott and Gottlieb, 1943; Hill, 1944; Sessions Hodge, 1945; Stafford-Clark et al., 1951; Hill and Pond, 1952). Based on these findings, a few researchers proposed classifications for certain sub-groups of psychopathy predicated on distinct wave patterns. Simons and Diethelm (1946, p. 622) observed that “psychopathic personalities with poor ethical standards and resulting social difficulties” exhibited “moderately slow activity,” which set them apart from other psychopaths. For the sake of simplicity, Kennard (1956, p. 109) separated “the pure and the aggressive psychopath, solely because these correspond to particular EEG patterns.”

Yet, all these attempts were provisional. EEG seemingly picked up abnormal signals from abnormal people, but the degree and type of abnormality varied (Cohn, 1949; Hill and Parr, 1963). Accordingly, some psychopaths had abnormal EEG others did not. This generated ideas about there being two types of psychopathic personality: one with normal and one with abnormal EEG (Knott and Gottlieb, 1944). Many experts conceded that the observation and identification of certain rhythms were of limited diagnostic utility: the EEG was merely a supplement—not a substitute—to psychiatric assessment (Diethelm and Simons, 1946; Silverman, 1947; Hill, 1952). Moreover, EEG-based classifications of psychiatric or criminal subgroups were speculative at best and often met with criticism. Psychiatric categories in general did not correspond to electrophysiological categories. Research results were irritatingly inconsistent. Researchers identified reasons for this incongruity in conceptual confusion and inadequate samples. Psychopathy was a wastebasket and delinquency a broad category. Samples consisted of diverse mental and neurologic patients as well as unequal criminals (Karpman, 1948; Hill, 1952; Kennard, 1953, 1956; McCord and McCord, 1956, 1964; Craft, 1966; Syndulko, 1978). The desire for an anatomical definition of psychopathy or delinquency could not be satisfied with the EEG (Cason, 1948; Sessions Hodge and Walter, 1953).

### “cerebral dysrhythmia”: EEG in etiology and therapy

Could EEG contribute to establish badness as dysfunction of genes, bodies, and brains in the 1940s? The potential of EEG lay in its capability to unveil brain function. If EEG curves indicated brain function, then—so the reasoning went—aberrant curves signified cerebral dysfunction. Yet, it was not known precisely how, where, and why “cerebral dysrhythmia” (see Hill, 1944) originated. Several researchers surmized an inborn defect as cause that was passed on through generations (Williams, 1941; Gottlieb et al., 1947; Knott et al., 1953). To others, EEG data made clear “that the psychopath possesses a brain which is malfunctioning, and which has been malfunctioning since early childhood” (Knott and Gottlieb, 1943; Silverman, 1943, p. 28). The undecided question whether deviant brain waves were signs of aberrant heredity, or of malfunctioning brains, or both, complicated matters regarding the etiology of badness. Explanations using EEG were often of rather general nature: experts associated “‘good’ personality and ‘good’ EEG” (Kennard, 1953, p. 104) or stability of brain waves with stability of personality (Ehrlich and Keogh, 1956). Explanations of psychopathy in particular referred to “disturbed cortical function” (Silverman, 1944b, p. 439), “defect in physiologic functioning” (Diethelm and Simons, 1946, p. 411), or “elasticity in […] neural limits” (Knott and Gottlieb, 1944, p. 519). Out of these rather broad attempts at etiology, two more concrete theories emerged that testified to the role of EEG as theory inspiring tool (Cf. Borck, 2005a, p. 264).

Psychopaths' childlike impulsivity, their lack of forethought and restraint along with the fact that many bearing the label were in their early twenties suggested that the condition consisted in a failure to mature (Mangun, 1942). In addition, the EEG curves of psychopaths resembled those of children, whereas normal adults' brain waves followed a distinct pattern. Thus, cortical immaturity mirrored behavioral immaturity, suggesting that psychopaths' brains were underdeveloped. The observation that the peculiar EEG abnormalities decreased with age corroborated the so-called cortical immaturity hypothesis (Hill and Watterson, 1942; Silverman, 1944a; Schwab, 1951; Hill, 1952). With regard to therapy, this indicated that anti-social, criminal, and immoral behavior was arrested development and that it cured itself through aging. Just as the cortical immaturity hypothesis, the second relevant theory of the day was based on a similarity of the brain waves of two distinct groups of people. By visualizing dysrhythmia, the EEG had proven to be a useful tool in the diagnosis of epilepsy. Dysrhythmia, or more generally, abnormal EEG also showed in the records of aggressive, criminal, and psychopathic people (Hill, 1944; Ostow and Ostow, 1946). The idea that “latent epilepsy” (see Brill and Walker, 1945) caused these behaviors, opened up new options for therapy. If anticonvulsive drugs alleviated epilepsy, they might also reduce aggression and anti-sociality. Correspondingly, behavior problem children, delinquents, and psychopaths were treated with anticonvulsants in explorative trials (Brown and Solomon, 1942; Silverman, 1944b). Yet, other experts soon contested the similarity of epilepsy and misbehavior because it owed its existence to a “regrettable fault of logic” (Sessions Hodge and Walter, 1953, p. 163). Dysrhythmia in the brain waves of epileptic and immoral people was not identical. Vague terminology had misled proponents of EEG in their suggestion for treatment (Hill and Pond, 1952; Sessions Hodge and Walter, 1953). Nevertheless, EEG contributed to the framing of immoral and unlawful behavior as neurobiological disorder: “The attitude that the psychopath may be a sick individual rather than a bad one receives some support from brain wave studies” (Brill and Walker, 1945, p. 549). Correspondingly, Silverman (1944a) called for changes in the medico-legal system and for special institutions for these seemingly irremediable individuals (on legal issues, see Conrad, 1959).

## Analysis: EEG, immorality, and methodological uncertainty

EEG research on immoral people's brain waves produced cursory and inconsistent findings. Breakthroughs were not attained. Researchers deliberated on contradictory results and review articles documented the heterogeneity of the findings (Kennard, 1953, 1956; Ellingson, 1954). Amidst the confusion, admonishing voices raised concerns regarding the utility of EEG in psychiatry and criminology (Walter, 1944; Gibbs and Gibbs, 1964). Simultaneously, an expert debate on the origin, production, meaning, and interpretation of the EEG took place in the 1940s and 1950s. In this context, the power of EEG as research tool and especially its scope of application were critically discussed. This methodological debate pervaded EEG research on unethical persons and is of great significance in understanding its historical trajectory. In what follows, I analyze methodological and theoretical issues that impeded the acuity of vision of “the wondrous eyes” of the EEG.

### “a relatively new yardstick” and a mysterious signal

“Only very few objective facts stand out in the sea of confusion surrounding the concept of psychopathic personality. The brain wave pattern is such a fact. This pattern cannot be disguised or falsified.” (Ehrlich and Keogh, 1956, p. 286)

Were brain waves really objective, factual, precise, and valid? Parenthetically Gottlieb et al. (1945, p. 138) described EEG as “a relatively new yardstick.” This metaphor captured the uncertainty associated with the new technology in its early days. EEG as a “new yardstick” referred to two-fold novelty: a new technological device and a new measurement method. Firstly, the technological device differed between laboratories. Scattered laboratory technicians engineered their own electroencephalographs with diverging electrodes, channels, and amplifiers. In addition, the technology was constantly developed further in the 1940s and 1950s (Collura, 1993; Rösler, 2005). Gibbens et al. (1955, p. 131) commented on this issue in one of their follow-up studies on criminal psychopaths: “EEG technique has made great strides in the last 5 years; study by modern methods might have revealed much more valuable information.” Thus, EEG technology varied geographically and transformed in the period under review with unclear consequences for data production and comparability of findings (Kennard, 1953; Adrian, 1963/1950; Fabisch, 1966). Secondly, EEG data was mysterious and its relation to the mind nebulous (Borck, 2005b). It was generally agreed that the EEG signal reflected electrical activity of the nervous system, but the precise neurophysiological origin remained obscure (Jasper, 1948; Cohn, 1949; Walter, 1950; Schwab, 1951; Cobb, 1971). Additionally, the proportion of signal and noise in the brain wave was not entirely clear. Researchers criticized others for taking artifacts to be data (Walter, 1944). Also, the EEG signal was volatile: movement, as well as physiological and psychological states disrupted it. Eye-movement, blood sugar, epilepsy, flickering light, hyperventilation, age, relaxation, sleep—all of these and other determinants altered the brain waves. Some alterations were distinct and already classified; others were still being sorted out. The measurement tool was in the process of being calibrated. The resulting uncertainty impacted knowledge production. For example, Gibbs et al. (1945) qualified their initial statements on the incidence of abnormal EEG in criminals once they accounted for age as confounding variable. Hence, the “yardstick” was under permanent construction in the 1940s and 1950s.

### The art of interpretation

“[…] the interpretation of the EEG is still an art which must be learned through experience.” (Silverman, 1947, p. 74)

In addition to the unknown origin and partly indeterminate constitution of the EEG signal, its meaning was encrypted: brain waves were not self-explanatory. Rather they were a code in need of decipherment (Davis, 1938; Borck, 2005b, 2008). A classificatory and interpretative system needed to be devised. Accordingly, experts classified alternating amplitude and frequency as distinct wave patterns (e.g., theta-rhythm), related these rhythms to distinct physiological or psychological phenomena (e.g., sleep), and in so doing charged the electrical signals with meaning. The difficulty to make sense of EEG curves increased with the complexity of the phenomena studied. Complex psychological and social phenomena, such as delinquency, did not express themselves in simple wave patterns. Despite the growing availability of EEG data owing to the spread and refinement of the technology, interpretation remained demanding. This illustrates the essential difference between producing EEG data and making sense of them by means of interpretation. Making sense of brain waves, however, was saturated with subjectivity: “[i]n short, no two electroencephalographers interpret all EEG's in exactly the same manner” (Williams, 1941; Ellingson, 1954, p. 264; Syndulko, 1978). Thus, contradictory findings could result from researchers' bias, stemming from differences in experience, training and skill (Gibbs and Gibbs, 1941; Lindsley, 1944; Silverman, 1947). Moreover, the art of interpretation changed over time and was susceptible to fashions (Sessions Hodge and Walter, 1953; Cobb, 1971). Attempts to improve interpretation aimed at eradicating subjective elements. For example, over the years automated analysis procedures replaced the “naked-eye method of analysis” (Greenblatt and Sittinger, 1950, p. 313). Trained judgment thus was heterogeneous and was frequently considered as a source of bias that vitiated the objectivity of EEG research (Daston and Galison, 2007).

### Normality and abnormality in the making

“It is tantalising, at present, to see the elaborate records of electrical oscillations, to know that they are, in some measure at least, a picture of the activity of the human brain, but to be unable to interpret the picture except to say where it is grossly distorted.” (Adrian, 1963/1950)

Gross distortion was what EEG could detect; gross distortion compared to what? Gauging data as abnormal required a norm. Normality as a reference category, however, had to be ascertained. Around 1940, normal EEG was poorly defined and, hence, classifications of abnormality varied (Jasper et al., 1938; Williams, 1941; Bloch and Hill, 1982). Around a decade later, Hill (1952, p. 440) described the prevailing biases in earlier years with the statement: “[w]hat is abnormal to some is, still, normal to others.” In addition to the above described fickleness of the EEG signal, approximately 15 per cent of normal people had abnormal EEG. The reliability of EEG as indicator for brain dysfunction was in question. As a countermeasure, experts attempted to constitute normality. Multiple, partly contradictory classification systems and norming samples coexisted; an influential one being Gibbs and Gibbs' (1941) comprehensive *Atlas of Electroencephalography*. However, variability in normality continued to hamper research well into the 1950s, rendering earlier studies on immoral persons' abnormal brain waves disputable (Schwab, 1951; Sessions Hodge and Walter, 1953; Ellingson, 1954; Kennard, 1956; Hill and Parr, 1963). Along with the standards for normality, the standards for doing proper science with EEG were changing.

### Acknowledging problems, seeking remedies, and changing the rules

One of the most striking features of the early EEG research on psychopathy, delinquency, and immorality was the researchers' almost unanimous agreement on the associated methodological problems. Proponents of the new technology frequently highlighted the poor understanding of the EEG signal, the technological difficulties and idiosyncrasies in its production, the subjectivity in its interpretation, the arbitrariness of normality as reference category, the limits in EEG's diagnostic utility, the inadequacy of the samples, and the heterogeneity of the people and concepts studied. Moreover, they acknowledged the tentativeness of their findings and often presented their theorizing as mere speculation. For example, Sessions Hodge (1945, p. 472) opens a paper with the statement that it “should be considered a preliminary communication, tentative and suggestive.” The prevalent comments on the premature, provisional, and inchoate state of the research testified to its predominantly explorative nature. Reconnaissance appeared to be the main goal. In the process of exploration, researchers identified shortcomings. Knowledge production was somewhat insular and its means so diverse that results from different laboratories were often incommensurable (Kennard, 1953; Ellingson, 1954; Syndulko, 1978). Recognition of the putative problems allowed for tackling them. Accordingly, experts sought to remedy inconsistency, heterogeneity, and diversity. The aspired cure lay in unifying research methods: standardization was the key.

In the 1940s and 1950s, the scientific community launched extensive initiatives to standardize EEG research. There appeared to be great need in every associated area. In the first issue of the newly established Journal of Electroencephalography and Clinical Neurophysiology, Grey Walter (1949, p. 474) stated: “If we are to understand one another at all, our language must contain agreed terms and symbols, conventional signs and scales, yet must admit originality.” Experts, then, did not speak the same language. Terminological confusion continued to be a problem. Until 1961, even the most essential aspect of EEG, the wave, had not been defined (Brazier et al., 1961). The administration of anticonvulsive drugs to psychopathic criminals owing to a misinterpretation of the term dysrhythmia (see above) was a noteworthy consequence of the unstandardized language (see also Hill, 1952). Diverging electrode placement was another cause of dissimilitude identified in the late 1940s. As a countermeasure, committees suggested unifying the positions of the electrodes on the scalp (Jasper, 1958). Furthermore, the already mentioned attempts to establish norming samples to homogenize judgments on abnormality (e.g., Gibbs and Gibbs, 1941) and the introduction of automated data analysis procedures to reduce subjectivity in interpretation of data (Niedermeyer and Lopes da Silva, 1987) demonstrated how researchers scrutinized and changed the standards of the science in the period under review. Rules for data production, interpretation, and analysis were in transition: the calibration of EEG was on ongoing process.

### Conclusion on the past: “The wondrous eyes” of the EEG in historical perspective

The emergence of EEG in the 1930s had opened up an uncharted epistemic terrain; a frontier worth exploring. The anticipation was great. Unable to predict what seminal discoveries EEG might facilitate, experts applied the new research tool to all sorts of psychological phenomena.

Yet, they operated under uncertainty with the novel method. The science was in its infancy: brain waves were mysterious. Data accumulation was somewhat haphazard and rarely driven by hypotheses. The new research tool needed to be handled, understood, and gauged. Standards, rules, and guidelines for using EEG were only in the making and research proceeded in the absence of specified methodological criteria. The lack of knowledge and constraints created scientific freedom: in the early days of EEG research almost *anything went*. Simultaneously, a debate on the limits and potentials of EEG took place. The reliability, the validity, and the scope of application of EEG were being investigated, negotiated, and determined. Proponents of EEG acknowledged tentativeness, identified shortcomings, introduced remedies, and established criteria for doing proper science with the new technology in the process. Thus, knowledge production with EEG, calibration of EEG, and sense-making of psychopathic, delinquent, and immoral persons via EEG occurred simultaneously in the 1940s and 1950s.

In this period, “the wondrous eyes” of EEG wandered over immoral persons' brains without spotting significant characteristics. The findings were inconclusive. There were no comprehensive EEG-based theories that connected the results or satisfactorily explained human badness. The new technology failed to deliver the hoped for revelations regarding diagnosis, classification, etiology, and therapy. In general, the contribution of EEG to psychiatry proved disappointing (Walter, 1944; Schwab, 1951; Borck, 2008). In the 1960s, Gibbs and Gibbs (1964, p. 460) commented: “The electroencephalographic study of psychiatric disorders has yielded surprisingly little information. This does not mean that it is of no value, but the electroencephalographer should not deceive himself; his technique does not convert psychiatric disorder into an ‘open book’.” Consensus formed that the complexity of psychiatric disorder could not be reduced to brain waves. Accordingly, experts gradually attenuated their hopes and acknowledged that the immoral brain remained inscrutable and intractable despite the novel technological outlook. In hindsight, researchers attributed the perceived failure of EEG to methodological problems: ill-defined concepts, contaminated samples, wanting norming samples, unstandardized technology, unclear terminology, and cryptic data had disabled research (Ellingson, 1954; Hill and Parr, 1963; Craft, 1966; Syndulko, 1978). Rather than clarifying the picture, EEG had added to the obscurity by providing an alternative, yet distorted image of wickedness. After 1960 EEG in general was beyond its zenith (Niedermeyer and Lopes da Silva, 1987). The hype surrounding the new technology abated. Correspondingly, EEG research on unethical and unlawful persons lost momentum, though studies kept on appearing in the following decades (Volavka, 1987; Milstein, 1988; Raine, 1993; Dolan, 1994). Although there was no definite closure, researchers gradually neglected the brain waves of psychopaths and delinquents and pursued the study of other psychophysiological measures (e.g., skin conductance and heart rate) (Hare, 1970). Today, EEG research continues, but shares the observation of psychopathy, delinquency, and immorality with the “wondrous eyes of a new technology.”

## Comment on the present: a novel set of eyes

In 1997, what is believed to be the first neuroimaging study on psychopathy appeared (Intrator et al., 1997; see Hare, 2007). In 2001, the first functional magnetic resonance imaging (fMRI) studies on moral judgment followed (Greene et al., 2001; Moll et al., 2001). Up to the present, a host of neuroimaging research on antisocial persons has been conducted (Fumagalli and Priori, 2012). Neuroimaging provided a novel set of eyes and an auspicious perspective on the immoral brain. Again, the new sight promises new insights. The present situation in neuroimaging resembles the early days of EEG research in the 1940s. Needless to say, the technologies themselves, the research procedures, the produced data, the definitions of immorality and psychopathy, and the historical context differ markedly. However, certain noteworthy parallels exist that merit a comparison.

Few topics evaded the prying eyes of the new technologies. Experts used both to explore almost the entirety of the human mind, ranging from economic decision-making to romantic love (Abi-Rached and Rose, 2010; Gergen, 2010). The exploration of freshly accessible phenomena entailed unsystematic data collection in some areas with no apparent goal other than using the new technologies. Both technologies operate on the same tenet, stating that deranged, illicit, and unethical behavior can be understood by studying the brain. This belief also promoted the rise of biomedical experts, claiming specialist brain-based knowledge about vice and virtue (Becker, 2012). Furthermore, the emergence of both technologies sparked great hopes for the study of psychopathy, delinquency, and immorality, accompanied by rhetoric of promise that advertised their massive potential and, for a certain time, perpetuated their application despite the absence of ground-breaking discoveries. This rhetoric strategy is exemplified by experts' formulaic reservations that emphasize the tentativeness of the findings along with calls for more research found in almost every neuroscientific research report (e.g., Anderson and Kiehl, 2012). Just as in the past, vignettes for anti-social behavior continue to be equivocal, contributing to contaminated research samples and related issues of comorbidity (Mullen, 2007; Pickersgill, 2009; Müller, 2010). Analysis and interpretation of EEG and neuroimaging data was and is a versatile art, with different approaches yielding unequal results (Carp, 2012). The meaning and significance of the data regarding the assessment of misdemeanants was and is equivocal and contested. Over the years, EEG and neuroimaging were calibrated, validated, and refined, altering the quality of the produced data (Logothetis, 2008). These processes of calibration and changing styles of interpretation testify to the non-neutrality of brain-focused methods. Making sense of the generated data was and is a matter of human expertise defined by standards that changed in the past and that are likely to change in the future. Thus, neither EEG nor neuroimaging provide direct access to morality in the brain; the access is restricted and mediated at best (Schirmann, 2013b). Neuroimaging aided to “change the picture” (Borck, 2008, p. 377) without remedying the persistent methodological and theoretical shortcomings. By altering the view, neuroimaging has provided clues, but certainly no incontrovertible evidence. Hence, the immoral brain remains elusive in the present.

The commonalities between EEG and neuroimaging suggest that history might have a lesson to offer. In the past, EEG enabled a new perspective on psychopathy, delinquency, and immorality and facilitated hopes for imminent research breakthroughs. In the ensuing exploration of the power of the EEG, experts encountered constraints: their research tool was in need of calibration and its utility restricted. EEG illuminated certain neurologic phenomena (e.g., epilepsy), yet yielded negligible results in psychiatry (e.g., criminal psychopathy). Hence, experts assessed the limits and potentials of EEG and adjusted its scope of application, neglecting it in the study of complex psychological and social phenomena. In a similar vein, neuroimaging is—and should be—up for discussion at the moment. Reliability, validity, utility, and credibility of neuroimaging in the study of diverse psychological and social phenomena are not a given, but are in need of critical acclaim. While neuroimaging has doubtlessly revolutionized our understanding of brain functioning, it has failed to deliver more than provisional evidence in forensic psychiatry and criminology. Just as with EEG, a fair appraisal and an informed critique of neuroimaging are needed in order to illustrate its potentials and mark its limitations. In conclusion, “the wondrous eyes” of new research tools clearly enable novel perspectives, insights and visions—yet it needs to be determined when, where, and how they clarify sight or cloud the outlook.

### Conflict of interest statement

This article was supported by the grant ‘Intuition and Emotion in Moral Decision-Making: Empirical Research and Normative Implications’ by the Volkswagen Foundation [Az. II/85 063].
